# Long-term effects of BCG vaccination on telomere length and telomerase activity

**DOI:** 10.1016/j.isci.2025.113159

**Published:** 2025-07-18

**Authors:** Ozlem Bulut, Valerie A.C.M. Koeken, Simone J.C.F.M. Moorlag, Charlotte J. de Bree, Vera P. Mourits, Gizem Kilic, Priya A. Debisarun, Marijke P.A. Baltissen, Joost H.A. Martens, Jorge Domínguez-Andrés, Leo A.B. Joosten, Mihai G. Netea

**Affiliations:** 1 Department of Internal Medicine and Radboud Center for Infectious Diseases, Radboud University Medical Center, Nijmegen, the Netherlands; 2 Department of Molecular Biology, Faculty of Science, Radboud University, Nijmegen, the Netherlands; 3 Department of Medical Genetics, Iuliu Hațieganu University of Medicine and Pharmacy, Cluj-Napoca, Romania; 4 Department for Immunology and Metabolism, Life and Medical Sciences Institute (LIMES), University of Bonn, Bonn, Germany

**Keywords:** Immunology, Age, Cell biology

## Abstract

This study explores the effects of Bacillus Calmette-Guérin (BCG) vaccination on telomere maintenance, an aging-related process, in immune cells. While BCG reduces systemic inflammation and enhances innate immune responsiveness by inducing trained immunity, its effects on other immune aging hallmarks, such as telomere shortening, are not fully understood. We assessed telomere length in two independent human cohorts before and three months after BCG vaccination. Telomere shortening was consistently observed after BCG, but not after placebo vaccination. Trained immunity non-responders were likelier to lose telomere length, but only among males. Higher pre-vaccination testosterone levels were associated with greater telomere loss in males. *In vitro*, BCG training activated telomerase, particularly in females, and this was partially prevented by exogenous testosterone. These findings suggest BCG vaccination influences telomere dynamics in a sex-specific manner, contributing to understanding BCG’s broader effects on aging-related processes.

## Highlights

• The BCG vaccine modulates immune cell telomere dynamics in a sex-specific manner
• Shorter telomeres are observed three months after BCG vaccination in healthy adults
• BCG’s effects on telomeres are linked to the trained immunity response, mainly in males
• BCG activates telomerase mostly in females and is blocked by testosterone

## Graphical abstract

**Figure:**
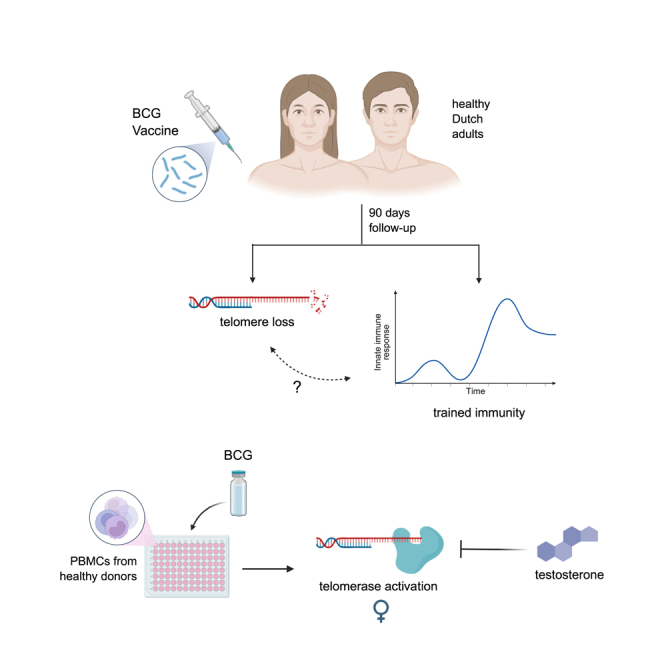

## Introduction

Innate immune cells can mount memory-like responses, a phenomenon termed “*trained immunity*”.^1^ This heightened response against nonspecific pathogens after exposure to certain vaccines and pathogen- or damage-associated molecular patterns (PAMPs and DAMPs) is orchestrated through epigenetic and metabolic reprogramming. One of the vaccines investigated in the context of trained immunity is Bacillus Calmette-Guérin (BCG),^2^ a live-attenuated *Mycobacterium bovis*, the pathogen that causes tuberculosis in cattle. Besides its use for tuberculosis prevention through Th1-based adaptive immune memory induction, BCG is considered a safe inducer of trained immunity in humans. Epidemiological studies and clinical trials have shown that BCG vaccination can reduce the incidence, severity, and mortality of heterologous infections.^3^^,^^4^

Aging of the immune system poses drastic problems as the world population rapidly ages. Older individuals suffer from higher morbidity and mortality due to their inability to mount a robust immune response during infections while responding poorly to vaccinations.^5^ Moreover, chronic inflammatory processes during aging exacerbate many diseases, such as diabetes, cardiovascular disease, obesity, cancer, and dementia.^6^^,^^7^ Over half of the individuals older than 65 have multiple age-related comorbidities.^8^^,^^9^ Importantly, immune aging is also directly related to the aging processes of solid organs.^10^ Tackling immune aging is, therefore, critical to reducing the disease burden on individual lives and healthcare systems. An aged immune system is characterized by persistent low-grade systemic inflammation and suboptimal cellular responses.^11^ Interestingly, BCG improves cellular immune responses while reducing systemic inflammation,^12^ and we hypothesized that it may impact other hallmarks of aging, one of which is telomere shortening.

Telomeres are repetitive TTAGGG sequences at the ends of eukaryotic chromosomes.^13^ A group of proteins bound to telomeres called the shelterin complex protects the chromosome ends from being recognized as DNA damage. However, shelterin and telomeres must unfold during replication to make the free end accessible to the replication machinery. With each replication cycle, telomeres are gradually eroded, and critical telomere shortening leads to cell-cycle arrest and is one of the hallmarks of cellular aging.^14^ Telomere length (TL) is affected by many extrinsic factors, including certain infections and total infectious burden.^15^^,^^16^^,^^17^^,^^18^ Oxidative stress, which often accompanies inflammation, can aggravate telomere shortening.^19^ Shorter telomeres are associated with many age-related conditions, including neurodegenerative diseases, atherosclerosis, and metabolic syndrome.^20^ However, abnormally long telomeres can also lead to clonal hematopoiesis and cancer through prolonged cell survival and accumulation of mutations.^21^ Proper regulation of TL at the cellular level is thus required for homeostasis of the organism.

Telomerase, a ribonucleoprotein consisting of an RNA template and a reverse transcriptase, can extend the telomeric repeats. The transcription of telomerase reverse transcriptase (TERT) is repressed in most adult human cells but is active in many cancers, allowing cancer cells to replicate excessively.^22^ Hematopoietic stem cells show telomerase activity during homeostasis, and T lymphocytes can upregulate it upon activation.^23^^,^^24^ Telomerase modulates NF-κB transcription,^25^ and NF-κB-p65, in return, facilitates the nuclear translocation of TERT, which is also regulated by TNF.^26^

In the present study, we revealed transcriptional regulation of telomere maintenance-related genes by *in vitro* BCG training and assessed TL before and three months after vaccination in two independent human BCG vaccination cohorts. In addition, we investigated the effects of BCG on telomerase activation *in vitro*.

## Results

### BCG transcriptionally regulates genes required for telomere maintenance in immune cells

**Figure 1:**
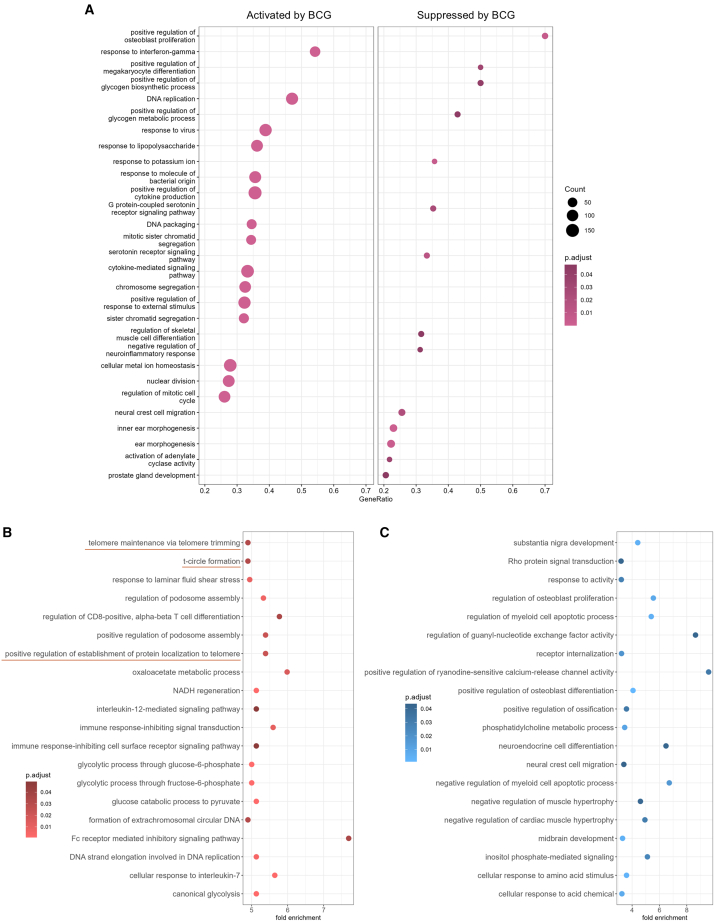
Transcriptional changes upon *in vitro* BCG training revealed an impact on telomere maintenance (A) Gene set enrichment analysis performed after RNA sequencing upon *in vitro* BCG training of healthy PBMCs (*n* = 5) compared to non-trained controls. (B and C) Overrepresentation analysis using the 3384 genes that were upregulated (B) and 2271 genes that were downregulated (C) by BCG. The top 20 significant (adjusted *p* < 0.05) biological processes with the highest fold enrichment are depicted. Processes related to telomere maintenance are underlined.

To investigate if BCG affected telomere maintenance, we first explored RNA sequencing data generated from 5 *in vitro* BCG-trained and control non-trained adherent PBMCs of healthy donors. 3384 transcripts were significantly upregulated, and 2271 were downregulated upon BCG training (adjusted *p*-value <0.05). Gene set enrichment analysis revealed many immunological processes activated by BCG, including response to interferon-gamma, response to virus, response to lipopolysaccharide, positive regulation of cytokine production, and cytokine-mediated signaling pathway (Figure 1A). Additionally, cell cycle-related processes such as DNA replication, mitotic sister chromatid segregation, nuclear division, and regulation of mitotic cell cycle were enriched by BCG training. Processes downregulated by BCG included positive regulation of osteoblast proliferation, positive regulation of megakaryocyte differentiation, positive regulation of glycogen biosynthetic process, positive regulation of glycogen metabolic process, response to potassium iron, G protein-coupled serotonin receptor signaling pathway, and negative regulation of neuroinflammatory responses.

Overrepresentation analysis using the PANTHER database revealed that multiple biological processes related to telomere maintenance were regulated by BCG training, along with immunological and metabolic processes (Figure 1B). Telomere maintenance processes such as “telomere trimming”, “t-circle formation”, and “positive regulation of establishment of protein localization to telomere” were significantly enriched in the genes upregulated by BCG training. Differentially expressed genes belonging to these processes and their fold changes are provided in Table 1. Among the genes downregulated by BCG, enriched processes included Rho protein signal transduction, regulation of osteoblast proliferation, regulation of myeloid cell apoptotic process, regulation of guanyl-nucleotide exchange factor activity, and neuroendocrine cell differentiation (Figure 1C).

**Table 1:** Differentially expressed genes belonging to the telomere-related biological processes enriched by BCG training *in vitro*

Gene	Full name	log2 fold change	*p* value	adjusted *p* value
**Positive regulation of protein localization to telomere**				

CCT5	Chaperonin Containing TCP1 Subunit 5	0,970563693	8,86028E-15	9,85354E-13
CCT6A	Chaperonin Containing TCP1 Subunit 6A	0,629685302	4,33981E-05	0,000499539
CCT3	Chaperonin Containing TCP1 Subunit 3	0,626906133	0,000151407	0,001410401
CCT8	Chaperonin Containing TCP1 Subunit 8	0,626826405	0,00014633	0,001372544
CCT7	Chaperonin Containing TCP1 Subunit 7	0,56957536	0,000186107	0,00167674
CCT2	Chaperonin Containing TCP1 Subunit 2	0,563903167	0,000392256	0,003094388
TCP1 (CCT1)	T-complex Protein 1 Subunit Alpha	0,536711745	0,000151239	0,001409439
CCT4	Chaperonin Containing TCP1 Subunit 4	0,501968204	4,60535E-05	0,000525364
DKC1	Dyskerin Pseudouridine Synthase 1	0,381099519	0,003041035	0,015956421
LARP7	La Ribonucleoprotein 7	0,25189321	0,005768843	0,026555537

**Telomere maintenance via telomere trimming/t circle formation**				

EXO1	Exonuclease 1	2,811564988	5,75955E-08	1,6327E-06
NBN	Nibrin	1,477106431	5,94306E-08	1,67414E-06
DNA2	DNA Replication Helicase/Nuclease 2	0,745845858	0,000604757	0,004383278
BLM	Bloom Syndrome Protein	0,718765946	3,77065E-06	6,26113E-05
XRCC3	X-ray Repair Cross Complementing 3	0,571177134	0,000358893	0,00286876
SLX4	SLX4 structure-specific endonuclease subunit	0,384836849	6,1397E-05	0,000668066
SLX1A	SLX1 homolog A, structure-specific endonuclease subunit	0,273331202	0,012927803	0,049672633
SMARCAL1	SWI/SNF Related, Matrix Associated, Actin Dependent Regulator Of Chromatin, Subfamily A Like 1	0,262165174	0,0032438	0,016824932

These transcriptional analyses supported our hypothesis that BCG vaccination may regulate telomere dynamics. To confirm this, we assessed the average TL before and up to 3 months after BCG vaccination in two independent cohorts of healthy adults.

### Sample selection from the 300BCG cohort

We selected equal numbers of trained immunity responders and non-responders from the 300BCG cohort to investigate BCG vaccination’s effect on TL. Trained immunity responders were defined as displaying at least a 20% increase in all three pro-inflammatory cytokines measured three months after BCG vaccination: IL-6, TNF, and IL-1β, upon *ex vivo* challenge with *S. aureus*. Non-responders were defined as showing no improvement in at least two pro-inflammatory cytokines 3 months after BCG vaccination. 40 young individuals were analyzed from each group. We only considered young participants between 18 and 35 to minimize the effect of age on TL. To also investigate the effect of sex, similar numbers of males and females were included in the responder and non-responder groups.

We also analyzed an older group of individuals between the ages of 50 and 71. The 300BCG cohort included only 24 individuals older than 50, and 20 had material available for TL measurements from all time points. Due to the smaller sample size, no responder/non-responder differentiation was performed for the individuals in the older group. Demographic details of all participants whose data were analyzed are presented in Table 2.

**Table 2:** Baseline characteristics of study participants whose telomere length was assessed

Study	Group	N (male/female)	Age (mean ± SD)	BMI (mean ± SD)
300BCG Study	Young responders	20/20	22,37 ± 2,74	22,68 ± 2,50
	Young non-responders	20/20	22,19 ± 1,65	22,69 ± 2,88
	≥50 years old	9/11	59 ± 7,40	24,86 ± 3,66
Validation Study	Placebo	1/4	22,80 ± 2,78	21,93 ± 3,56
	Single dose	7/6	25,31 ± 7,86	23,06 ± 2,29
	Double dose	5/8	22,08 ± 1,82	23,60 ± 2,32
	Revaccination	4/12	22,75 ± 2,82	22,88 ± 2,47

### BCG leads to shorter telomeres in healthy young individuals three months after vaccination

**Figure 2:**
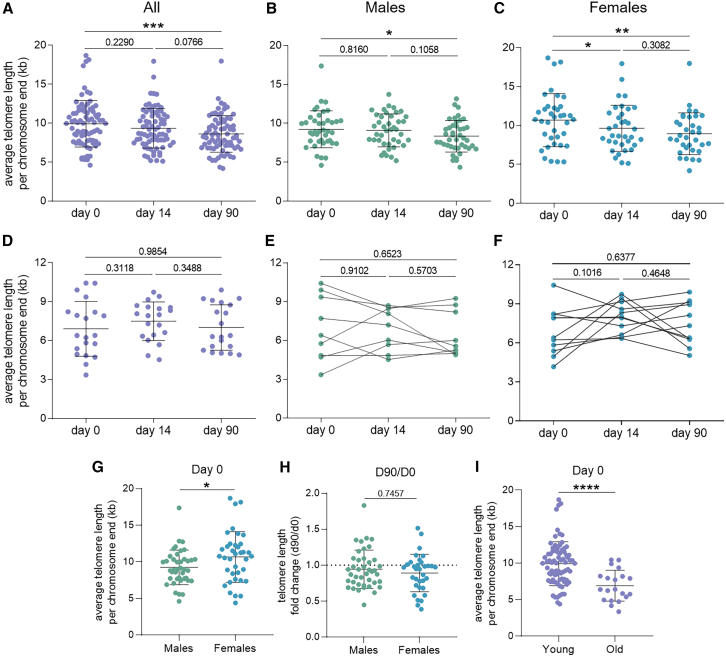
BCG vaccination led to shorter telomeres in young, healthy individuals 3 months after vaccination (A–F) Average telomere length (TL) per chromosome end in whole blood, quantified by qPCR, (A) in all young individuals (aged 18–35, *n* = 80), (B) young males (*n* = 40), or C. young females (*n* = 40) before, 14 days or 90 days after vaccination. Average TL in (D) all older individuals (aged 50–71, *n* = 20), (E) older males (*n* = 9), or (F) older females (*n* = 11) before, 14 days, or 90 days after vaccination. (G and H) Sex comparisons of (G) average TL before vaccination and (H) fold change in telomere length in three months. (I) Age group comparison of average TL before vaccination and fold change in TL in 3 months. Statistical analyses were performed using Wilcoxon matched-pairs signed rank test (A–F) or Mann-Whitney test (G–I). ∗*p* ≤ 0.05, ∗∗*p* ≤ 0.01, ∗∗∗*p* ≤ 0.001, ∗∗∗∗*p* ≤ 0.0001. Error bars indicate standard deviation.

When responders and non-responders were assessed together, the average TL of circulating cells was significantly lower for both sexes at three months (Figures 2A–2C). For females, this was already the case after two weeks (Figure 2C). Importantly, telomere shortening was not observed in every participant; some experienced no change or even underwent telomere elongation. 20 out of 42 males and 15 out of 38 females experienced more than 10% telomere shortening over three months (mean loss 20.0 ± 13.0% for males, 23.7 ± 19.2% for females). In the older group, no significant change was observed overall or in participants of either sex (Figures 2D–2F).

Notably, females had longer telomeres before vaccination than males (Figure 2G), but the average TL change in the 3 months after BCG was comparable between males and females (Figure 2H). Furthermore, older individuals had shorter average TL than young individuals before vaccination, which aligns with age-dependent telomere shortening (Figure 2I). The mean TL per chromosome end of old participants was 30.3% shorter than the mean of young participants. Overall, these results suggest that BCG vaccination can lead to telomere shortening in young individuals but not in individuals over 50 with shorter telomeres at baseline.

### BCG-induced telomere shortening is linked to the trained immunity response in males

**Figure 3:**
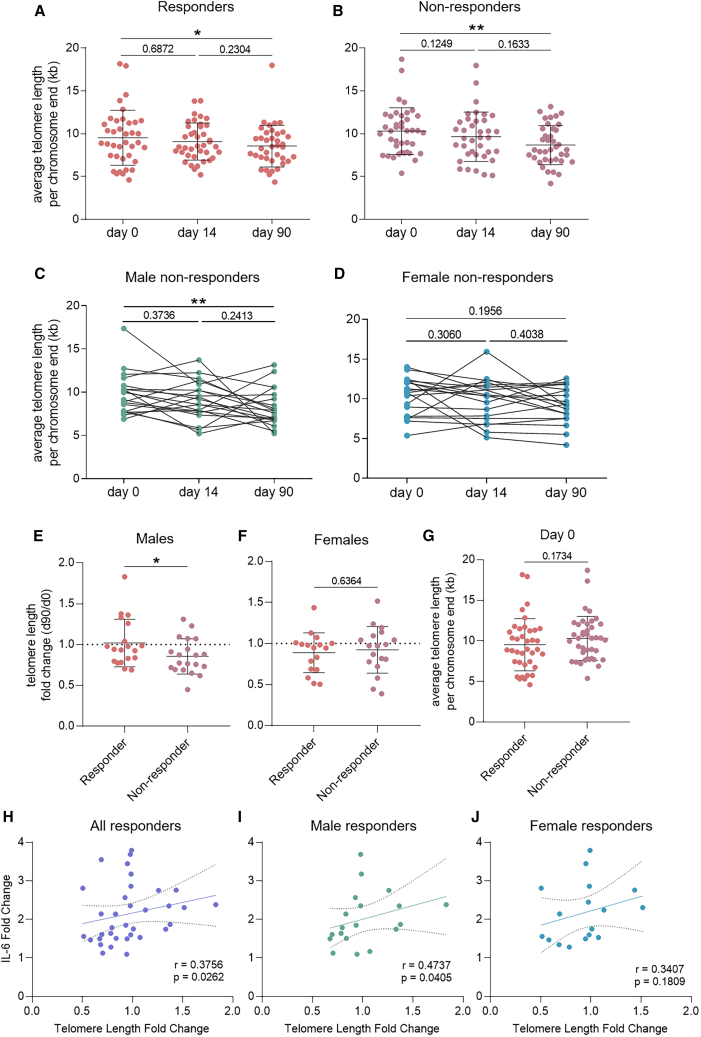
Telomere shortening was linked to the trained immunity response, particularly in males (A and B) Average telomere length (TL) per chromosome end in whole blood in young (aged 18–35) (A) responders (*n* = 40) and (B) non-responders (*n* = 40) before, 14 days, or 90 days after vaccination. (C and D) Average TL of (C) male (*n* = 20) and (D) female (*n* = 20) non-responders before, 14 days, or 90 days after vaccination. (E and F) Comparisons of the fold change in TL in three months for responders and non-responders among (E) males and (F) females. (G–I) (G) Average TL comparison of responders and non-responders before vaccination. Correlation of the change in IL-6 response and TL change over 3 months in (H) all, (I) male, and (J) female responders. Statistical analyses were performed using Wilcoxon matched-pairs signed rank test (A–D), Mann-Whitney test (E–G), or Spearman’s rank correlation (H–J). ∗*p* ≤ 0.05, ∗∗*p* ≤ 0.01. r: Spearman’s rank correlation coefficient. Error bars indicate standard deviation in A-B and E–G. Dashed lines in H-J indicate 95% confidence intervals.

Next, we investigated the effect of BCG on TL depending on the trained immunity responder status. Interestingly, stronger telomere shortening three months after vaccination was observed in non-responders (mean loss 24.3%, Figure 3B) compared to responders (mean loss 16.3%, Figure 3A). When non-responder males and females were analyzed separately, male non-responders had shorter telomeres after three months (Figure 3C), while it did not reach statistical significance for females (Figure 3D). However, the lack of significance in women may be only due to a lack of statistical power. TL change 3 months after vaccination differed significantly for male non-responders and responders, telomere loss being more pronounced in non-responders (Figure 3E). No differences between responders and non-responders were observed in females (Figure 3F). Of note, the average TL of responders and non-responders was not different before vaccination (Figure 3G). For responders, the improvement of *ex vivo* IL-6 response was correlated with telomere elongation (Figure 3H), but not TNF and IL1β (Figure S2). The IL-6 association was stronger and only significant in males (Figures 3I and 3J). These results suggest that telomere shortening after BCG vaccination is partly linked to the trained immunity response, particularly in males.

### Placebo-controlled validation study confirms BCG-induced telomere shortening

In order to validate our observations, we used samples from an independent clinical study in which participants were vaccinated with a placebo or BCG, and blood was collected before and 3 months after each intervention. The study aimed to test different regimens of BCG, including double-dose vaccination and booster vaccination.^27^ Details of the different study arms are provided in Figure S1.

**Figure 4:**
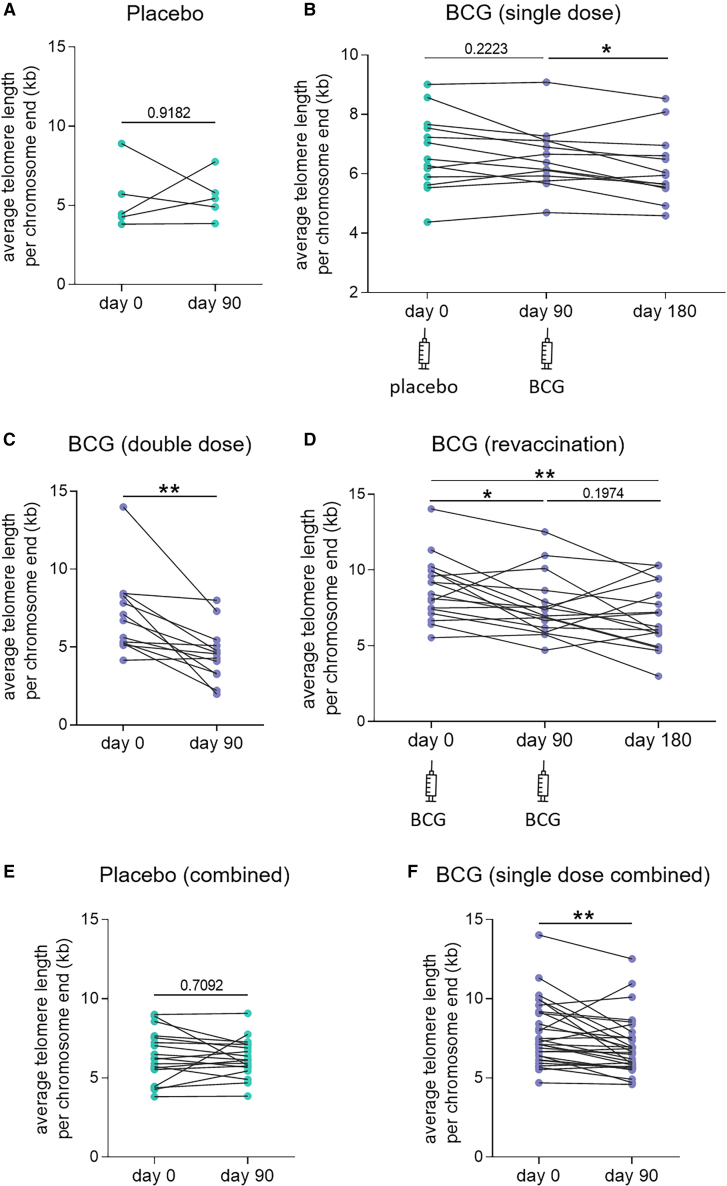
Telomere shortening 3 months after BCG vaccination in an independent validation cohort (A–D) Average telomere length (TL) per chromosome end in whole blood was assessed for different treatment arms: (A) placebo (*n* = 5), (B) single-dose BCG (*n* = 13), (C) double-dose BCG (*n* = 13), and (D) BCG revaccination (*n* = 16). (E) Average TL of all participants vaccinated with placebo and assessed before and 90 days after vaccination (*n* = 18). (F) Average TL of all participants vaccinated with one single-dose BCG and assessed before and 90 days after vaccination (*n* = 29). Statistical analyses were performed using the paired t test. ∗*p* ≤ 0.05, ∗∗*p* ≤ 0.01.

There was no clear trend for the 5 individuals in the placebo-only group (Figure 4A). The 13 participants of the single vaccination group also received a placebo at first and a BCG vaccination three months later, after which they were followed for another three months. It was evident in this group that placebo vaccination did not affect TL, while BCG vaccination led to shorter telomeres after three months (Figure 4B). The double-dose BCG group also exhibited shorter average TL after 3 months, with a mean loss of 35.4 ± 21% (Figure 4C).

In the revaccination group, the first BCG vaccine led to shorter telomeres after 3 months (Figure 4D). These individuals then received a second BCG. Three months later, the average TL was still significantly lower than the baseline. There was a trend toward further telomere loss after the booster vaccination, however, the difference before and 3 months after the booster was not statistically significant. Some individuals recovered their TL in this period.

When measurements of the single vaccination group before and after placebo vaccination were combined with the placebo group to increase statistical power, there was still no difference in TL after placebo vaccination (Figure 4E). When the first two measurements of the revaccination group and the last two of the single vaccination group were combined, the mean telomere loss linked to a single BCG was 31.7 ± 10.8% (Figure 4F).

In light of the consistent observations with BCG, we explored whether other trained immunity-inducing vaccines, such as the measles, mumps, and rubella (MMR) vaccine,^28^^,^^29^ would have similar effects on TL. Using material from a previous study by our group,^29^ we could assess TL in 8 healthy adults before and 30 days after MMR vaccination. Due to the limited sample size and the shorter follow-up period, the comparison did not reach statistical significance (Figure S3A). However, 7 out of 8 participants had shorter telomeres after vaccination. This data indicates that telomere shortening might not be specific to BCG vaccination but more general for other vaccines as well. The impact of other vaccines and whether the effects are long-term must be investigated in detail by dedicated studies.

### BCG activates telomerase, which is counteracted by testosterone

**Figure 5:**
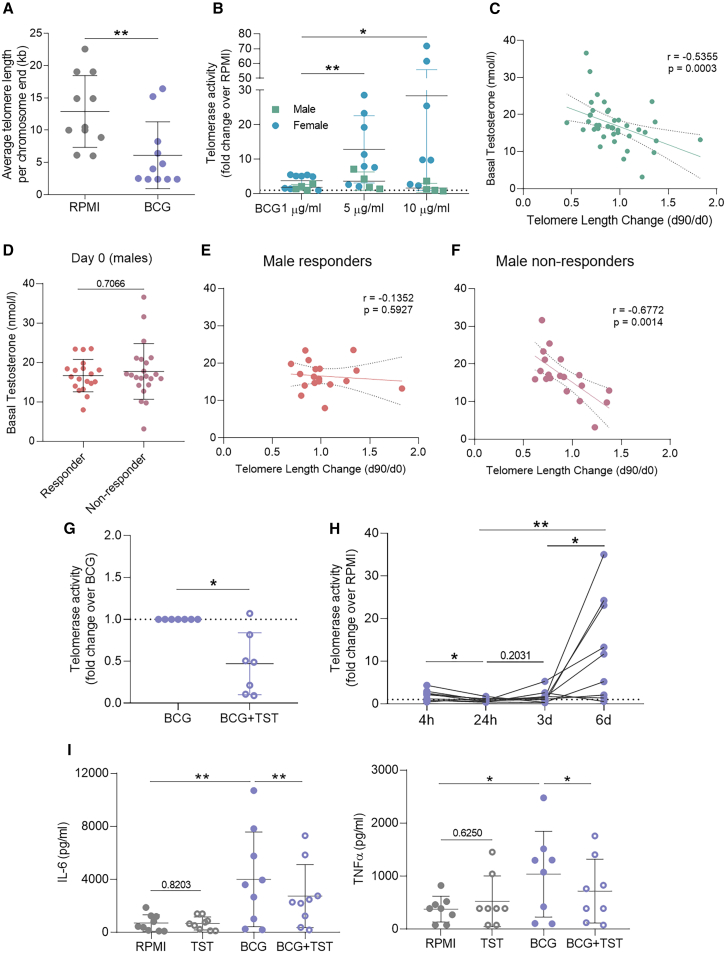
BCG-induced telomerase activation that can be inhibited by testosterone (A and B) (A) Average telomere length (TL) per chromosome end and (B) telomerase activity, represented as activity relative to RPMI control, at day 6 of *in vitro* BCG training of healthy PBMCs (*n* = 11–12). BCG concentration was 5 μg/mL for A. (C) Correlation of TL change in three months after BCG vaccination and basal testosterone levels in young males of the 300BCG cohort with available hormone measurements. (D–F) (D) Basal testosterone levels of male responders and non-responders of the 300BCG cohort. Correlation of TL change in three months with the basal testosterone levels in male (E) responders and (F) non-responders. (G) Relative telomerase activity of PBMCs from healthy female donors trained with 5 μg/mL BCG with or without 50 μM testosterone (*n* = 7). (H) Relative telomerase activity of BCG-trained female PBMCs compared to non-trained controls at different time points during the training protocol (*n* = 9). (I) IL-6 and TNF production by female PBMCs trained with BCG with or without testosterone upon restimulation with 10 ng/mL LPS (n = 8–9). Statistical analyses were performed using Wilcoxon matched-pairs signed rank test (A–B, G–I), Mann-Whitney test (D), or Spearman’s rank correlation (C, E–F). r: Spearman’s rank correlation coefficient, TST: testosterone, h: hour, d; day. ∗*p* ≤ 0.05, ∗∗*p* ≤ 0.01. Error bars indicate standard deviation in (A–B, D, G, and I). Dashed lines in (C and E–F) indicate 95% confidence intervals.

To assess BCG’s potential effect on telomerase activation, we used the established *in vitro* trained immunity model. BCG-induced telomere shortening was also observed in the *in vitro* setting, even to a greater extent, validating the observations *in vivo* (Figure 5A). Notably, the cytokine TNF also led to shorter telomeres in the training setting (Figure S3B), which aligns with other observations.^30^

We next assessed telomerase activity at the end of the training period to explore whether telomerase is activated to maintain a healthy TL. BCG training dose-dependently promoted telomerase activation (Figure 5B). Increased telomerase activity could also be observed to a lower extent with *C. albicans-*derived β-glucan, another well-known trained immunity inducer (Figure S3C).

Interestingly, BCG-induced telomerase activation was more prominent for female blood donors (Figure 5B). Although sex hormones are not present in physiological levels in the cell culture, cells can be epigenetically pre-programmed based on the *in vivo* hormone concentrations as demonstrated by recent research.^31^^,^^32^ To understand if the observed sex difference was related to hormone concentrations, we correlated the TL change to hormone concentrations at baseline in the 300BCG cohort, for which these measurements were available. Female participants had no or very low detectable levels of testosterone. Among males, baseline testosterone concentrations were strongly negatively correlated with the fold-change of TL after BCG vaccination (Figure 5C). No association was observed between TL change and androstenedione, dehydroepiandrosterone sulfate, or 17-hydroxyprogesterone concentrations (Figure S4).

Importantly, testosterone concentrations before BCG vaccination were not different between male responders and non-responders (Figure 5D). However, trained immunity responder males seemed to be protected against testosterone’s impact on TL (Figure 5E), while higher testosterone levels were strongly linked to more telomere loss (Figure 5F).

To further validate the role of testosterone, we added exogenous testosterone to PBMCs from healthy female donors during BCG training and assessed telomerase activation after training. Pre-incubation of the female cells with testosterone partially, and for some donors almost completely, prevented BCG-induced telomerase activation (Figure 5G). Assessing the BCG-induced telomerase activity at different time points during training revealed that activation mainly occured between days 3 and 6 post-BCG, days after a limited transient activation in the first hours after stimulation (Figure 5H). Notably, testosterone also partially suppressed the trained immunity response induced by BCG *in vitro*, measured as IL-6 and TNF secretion upon secondary challenge with LPS (Figure 5I). These results suggest a testosterone-dependent inhibition of the effects of trained immunity induction on telomerase activation.

## Discussion

Our data reveal that BCG vaccination leads to telomere shortening sustained for at least 3 months after vaccination in young adults below 35 but not in adults over 50 who have shorter baseline telomeres. We validated BCG-induced telomere shortening in an independent *in vivo* placebo-controlled study and an *in vitro* trained immunity model.

Possible mechanisms through which BCG leads to telomere loss include enhanced TNF and reactive oxygen species (ROS) production known to be induced by BCG-stimulated innate immune cells. TNF can cause telomere shortening, as also shown in Figure S3B,^30^ and ROS can damage the G-rich telomeric sequence, which is more vulnerable to oxidative damage than non-telomeric DNA.^33^ However, in the 300BCG cohort, TNF response did not correlate with the change in TL. Instead, the improvement of the IL-6 response was linked to protection from telomere loss. Our data also suggests that the MMR vaccine, another trained immunity-inducing live vaccine, could lead to telomere shortening (Figure S3A). However, since the follow-up period was shorter than the BCG studies and the sample size was limited, dedicated studies on MMR or other vaccines with 3-month or longer follow-ups are needed to clarify how generalizable vaccine-induced telomere damage is.

Notably, the two clinical studies used different strains of BCG: BCG-Bulgaria and BCG-SSI Denmark. Over 20 distinct BCG strains are used worldwide,^34^ and they have different immunostimulatory properties.^35^ Compared to BCG-Bulgaria, BCG-Denmark has more viable colony-forming units (CFUs) and is superior in stimulating cytokine production when tested in equal CFUs. Furthermore, a randomized controlled study with infants demonstrated higher specific T cell responses induced by BCG-Denmark than BCG-Russia and Japan.^36^ BCG strains have not been directly compared regarding their potency to induce trained immunity *in vivo*. However, it is plausible that the extent of immune activation is related to the degree of telomere damage. Although we observed telomere shortening with both BCG strains, the ratio of people experiencing telomere shortening and the average degree of loss was higher with BCG Denmark. Further research should directly compare BCG strains regarding their impact on telomere length and telomerase activity, as this might have implications for long-term risks and benefits.

We also showed the regulation of telomere dynamics by BCG Denmark at the transcriptional level. BCG upregulated multiple subunits of the chaperone TCP-1 Ring Complex (TRiC) (Table 2). TRiC is required for the folding of TCAB1, an essential component of the telomerase holoenzyme complex, and proper localization of telomerase.^37^^,^^38^ Dyskerin Pseudouridine Synthase 1 (DKC1) was also upregulated upon BCG training. This protein is another component of the telomerase complex. It binds to the RNA component of telomerase (hTR) and prevents its degradation.^39^ LARP7, upregulated by BCG, also protects against hTR degradation and is necessary to stabilize the interaction of hTR with hTERT.^40^ Thus, BCG transcriptionally activates processes that regulate telomerase assembly, localization, and stabilization.

When telomeres are over-elongated, such as in cancer cells, a trimming process occurs to control TL.^41^ This leads to the generation of circular telomeric repeats called t-circles. Lymphocytes were shown to activate telomere trimming to counter the heightened telomerase activity induced by phytohemagglutinin stimulation.^42^ BCG training upregulated several proteins implicated in this process including exonuclease 1 (EXO1) and helicase bloom syndrome protein (BLM) which are important for the resolution of telomeric G-quadruplex structures,^43^^,^^44^ nibrin (NBN), part of the MRE11-RAD50-NBN complex regulating telomere homeostasis,^45^ SLX1A and SLX4 whose recruitment at the telomeres helps maintain telomere integrity,^46^ and SMARCAL which protects telomere integrity during replication.^47^ Overall, in addition to telomerase regulation, BCG transcriptionally modulates telomere trimming and maintenance.

In the *in vitro* model of trained immunity, BCG dose-dependently activated telomerase, in line with the transcriptional results. Strikingly, this effect was primarily observed in immune cells from female donors. Circulating testosterone concentrations, but not other hormones, before BCG vaccination correlated with the change in TL three months later. The higher basal testosterone concentrations were, the more telomere loss occurred after BCG vaccination. Higher testosterone concentrations in men were previously linked to shorter telomeres in a large study.^48^ Furthermore, treatment of PBMCs from female donors with exogenous testosterone significantly prevented BCG-induced telomerase activation, supporting a role for testosterone in telomere attrition. Bioavailable testosterone sharply declines as males age.^49^ This might partly be why older participants did not experience telomere loss. However, it is also possible that cellular senescence is triggered to protect critical levels of telomere damage. Future studies seeking to replicate our results in older individuals should also measure senescence markers.

The degree of trained immunity induction is heterogeneous among people.^50^ In this study, we considered the group of people who displayed 20% or more improvement in cytokine production three months after vaccination as “responders”. Despite having similar baseline TL, trained immunity responders could maintain it better than non-responders, but this difference only existed among males. Lower baseline testosterone was also linked to better telomere maintenance for non-responders. Despite having similar testosterone levels, responder males did not exhibit a relationship between testosterone and TL change. Notably, testosterone partially inhibited the trained immunity response induced by BCG *in vitro.* In addition, the amplification of telomerase activity needed several days to be induced, similar to trained immunity,^51^ possibly owing to the epigenetic and metabolic reprogramming induced by BCG. Together, these support the hypothesis that the impact of BCG vaccination on telomere dynamics is linked to the capacity to establish trained immunity. The intriguing role of testosterone in this process warrants further investigation.

**Figure 6:**
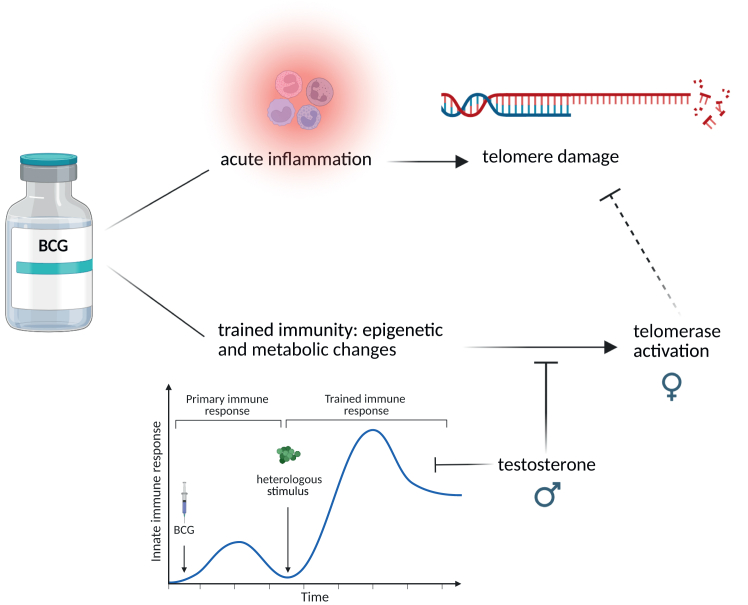
Schematic representation of telomeric regulation by BCG vaccination BCG leads to telomere damage due to the induction of initial acute inflammation, potentially through increased oxidative stress. On the other hand, BCG can induce long-term telomerase activation, likely linked to the establishment of trained immunity, and limit the telomeric damage induced by acute inflammation. Testosterone inhibits BCG-induced telomerase activation.

A working model of BCG’s effects on TL and telomerase activity is depicted in Figure 6. Through acute inflammation, BCG could lead to telomere loss. In parallel, however, BCG exerts long-term effects on trained immunity and telomerase activity, which may partially counteract telomere loss. Mechanisms responsible for long-term telomerase activation by BCG remain to be elucidated. Testosterone can inhibit BCG-induced telomerase activation and the trained immunity response. Male non-responders experienced more telomere loss than responders, and improvement of IL-6 response correlated with a positive TL change. Thus, the epigenetic and metabolic rewiring responsible for the induction of trained immunity may be involved in BCG’s effects on telomerase activation. Non-responders might be unable to maintain TL due to low telomerase activity, and male non-responders would be less likely to activate telomerase due to endogenous testosterone. Whether telomerase activation persists and eventually restores telomere loss *in vivo* remains to be studied.

Another intriguing possibility is that sustained effects of BCG on telomere dynamics are also induced at the level of bone marrow progenitors. This would explain our observations in circulating blood cells three months after vaccination, considering that most innate immune cells have shorter lifespans. BCG has already been shown to transcriptionally reprogram hematopoietic stem and progenitor cells (HSPCs) in the bone marrow.^52^ Therefore, assessing TL in HSPCs before and after BCG vaccination is warranted.

In conclusion, this study reports the long-term sex-specific effects of BCG vaccination on telomere maintenance and telomerase activity in healthy humans. To further tease out the underlying mechanisms in the absence of environmental microbial exposures, future studies may consider BCG vaccination studies in germ-free animals in a controlled environment. Single-cell analyses are necessary to understand which types of cells in circulation are most affected and the potential consequences of telomere shortening in these cells. Future studies should also address the impact of BCG vaccination on other hallmarks of aging, such as mitochondrial dysfunction and changes in DNA methylation. How the cellular effects of BCG vaccination may be translated into clinical outcomes is yet to be studied. Nevertheless, our results highlight an interesting relationship between BCG-induced trained immunity and cellular aging that warrants deeper exploration.

### Limitations of the study

Our study has some limitations to address. First, the low number of study participants over 50 years of age prevented sex-specific analyses in this subgroup of individuals, and future studies should reassess these effects in older individuals. Second, replicative telomere attrition does not have a linear relationship with the number of cell divisions. The erosion rate accelerates as cells age and depends on the initial TL.^53^ Therefore, further assessing BCG’s effects in older individuals with shorter baseline telomeres is warranted. Third, the shortest telomere is a better determinant of senescence than the average TL,^54^ but only the average TL was measured in this study as a proxy for cellular aging. More sensitive TL measurements such as quantitative fluorescence *in situ* hybridization (Q-FISH)^55^ should be performed in future studies. We preferred the widely used qPCR method due to the protocol’s high throughput and easy nature. However, with this method, it is only possible to determine an average TL, disregarding the heterogeneity between cell types and differentiation stages.

## Resource availability

### Lead contact

Any further requests for information, resources, and reagents should be directed to the Lead Contact Mihai Netea (mihai.netea@radboudumc.nl).

### Materials availability

This study did not generate new reagents or materials.

### Data and code availability

• RNA sequencing data is deposited to the Gene Expression Omnibus with the accession number GEO:GSE300272. Data of the 300BCG cohort, including cytokine production, have been used in previous publications and are available via the link https://doi.org/10.5281/zenodo.10288920. Telomere length measurements of both cohorts are provided in Files S1 and S2.
• Analysis pipeline for RNA sequencing could be reached from https://github.com/vanheeringen-lab/seq2science.
• Any additional information required to reanalyze the data will be made available by the lead contact upon reasonable request.

## Acknowledgments

J.D.-A. was supported by a Veni grant (09150161910024) of the Netherlands Organization for Scientific Research. M.G.N. was supported by an ERC Advanced Grant (833247) and a Spinoza grant from the Netherlands Organization for Scientific Research. Finally, the authors thank all clinical study participants and blood donors for their contributions.

## Author contributions

Conceptualization, O.B., J.D.-A., and M.G.N.; methodology, O.B., J.H.A.M., J.D.-A., and M.G.N.; formal analysis, O.B. and M.P.A.B.; investigation, O.B., V.A.C.M.K., S.J.C.F.M.M., C.J.d.B., V.P.M., G.K., P.A.D., and M.P.A.B.; resources, J.H.A.M., L.A.B.J., and M.G.N.; data curation, O.B., V.A.C.M.K., and M.P.A.B.; writing (original draft), O.B., writing (review and editing), V.A.C.M.K., S.J.C.F.M.M., and C.J.d.B., V.P.M., G.K., P.A.D., M.P.A.B., J.H.A.M., L.A.B.J., J.D.-A., and M.G.N.; visualization, O.B.; supervision, J.D.-A. and M.G.N.; project administration, O.B. and M.G.N.; funding acquisition, J.D.-A. and M.G.N.

## Declaration of interests

M.G.N. is a scientific founder of Trained Therapeutix Discovery (TTxD), Biotrip, Lemba Therapeutics, and Salvina Therapeutics. L.A.B.J. is a scientific founder of TTxD, Lemba Therapeutics, and Salvina Therapeutics.

## STAR★Methods

### Key resources table

**Table:** 

REAGENT or RESOURCE	SOURCE	IDENTIFIER
**Bacterial and virus strains**		

Bacille Calmette-Guérin vaccine	Intervax	Bulgaria strain
Bacille Calmette-Guérin vaccine	Statens Serum Institut	Danish strain
Heat-killed *Staphylococcus aureus*	Gift	Clinical isolate

**Biological samples**		

buffy coats from healthy adults	Sanquin blood bank	
whole blood from healthy adults	Radboudumc	Study participants
serum from healthy adults	Radboudumc	Donors with informed consent

**Chemicals, peptides, and recombinant proteins**		

Lipopolysaccharide (LPS)	Sigma-Aldrich	From *E. coli* serotype 055:B5, L2880
Ficoll-Paque PLUS	Cytiva	Cat#17144003
RPMI 1640 Medium (Dutch modification)	Gibco	Cat#22409031
Recombinant Human TNF (carrier-free)	Biolegend	Cat#570106
Testosterone	Sigma-Aldrich	Cat#T1500

**Critical commercial assays**		

Absolute Human Telomere Length Quantification qPCR Assay Kit	ScienCell	Cat#8918
Telomerase Activity Quantification qPCR Assay Kit	ScienCell	Cat#8928
Human IL-1 beta/IL-1F2 DuoSet ELISA	R&D Systems	Cat#DY201
Human TNF-alpha DuoSet ELISA	R&D Systems	Cat#DY210
Human IL-6 DuoSet ELISA	R&D Systems	Cat#DY206

**Deposited data**		

Cytokine production data from the 300BCG cohort	Human Functional Genomics Project	Data available at https://gitlab.com/xavier-lab-computation/public/bcg300
RNA sequencing data	This manuscript	GEO:GSE300272

**Experimental models: Organisms/strains**		

*In vitro* trained immunity model with human PBMCs	Donors with informed consent	Adapted from https://doi.org/10.1016/j.xpro.2021.100365
*In vivo* trained immunity model: the 300BCG study	Donors with informed consent	Part of Human Functional Genomics Project

**Software and algorithms**		

Prism 8	GraphPad Software	
R 4.1.3	The R Foundation for Statistical Computing	
R Studio 2023.09.1	Posit Software PBC	
clusterProfiler package	https://bioconductor.org/packages/release/bioc/html/clusterProfiler.html	
rbioapi package	https://cran.r-project.org/web/packages/rbioapi/index.html	
tidyverse package	https://cran.r-project.org/web/packages/tidyverse/index.html	

### Experimental model and study participant details

#### Study subjects and interventions

In the 300BCG study^50^^,^^56^ 323 healthy adult volunteers, including both males and females, were recruited between April 2017 and June 2018 at Radboudumc in the Netherlands and vaccinated with a standard dose of BCG (Bulgaria strain, InterVax) administered intradermally. Venous blood was collected before, two weeks after, and three months after vaccination. Exclusion criteria for the study were any vaccination within three months before inclusion, acute illness four weeks before inclusion, immunodeficiency or any other chronic disease, use of systemic medication other than oral contraceptives, use of antibiotics three months before inclusion, previous BCG vaccination, history of tuberculosis, previous contact with tuberculosis patients, being born in a tuberculosis endemic country, ancestry other than Western European, pregnancy, and breastfeeding. Participants were excluded if they used medication or were ill during the follow-up period.

As the validation cohort, we used the BCG-Booster randomized placebo-controlled trial assessing different BCG vaccination regimens.^27^ Recruitment of 51 healthy adult males and females of Western European ancestry took place between October 2019 and February 2020 at Radboudumc. Exclusion criteria included previous BCG vaccination, any vaccination three months before the study, plans for any vaccination during the study, acute illness within two weeks before the study initiation, and medication use, including non-steroidal anti-inflammatory drugs and excluding oral contraceptives, less than four weeks before the start of the study. Participants were allocated to receive a placebo vaccination (BCG vaccine diluent), a single standard dose of BCG (0.1 mL, 0.75 mg/mL, Denmark strain, AJ Vaccines), a high dose of BCG (0.1 mL 1.5 mg/mL), or revaccination with a single standard dose three months after the first vaccination. Groups received placebo vaccinations to align with the revaccination group. Blood was collected before and three months after each intervention. A schematic representation of the vaccination and blood collection regimens is provided in Figure S1.

Both studies were approved by the Arnhem-Nijmegen Medical Ethical Committee (NL58553.091.16 and NL58219.091.16 in the Dutch trial registry). Written informed consent was obtained from all participants.

#### *In vitro* trained immunity model with healthy human peripheral blood mononuclear cells (PBMCs)

Buffy coats from healthy male and female donors were obtained from the Sanquin blood bank. Donors gave informed consent for their material to be used for research purposes. PBMCs were isolated using Ficoll-Paque (GE Healthcare) density gradient separation and washed twice with phosphate-buffered saline (PBS). Cells were resuspended in Dutch-modified RPMI 1640 medium (Invitrogen), supplemented with 50 μg/mL gentamycin, 2 mM Glutamax (Gibco), and 1 mM pyruvate (Gibco). The *in vitro* trained immunity model was previously described in detail by Dominguez-Andres et al.^57^ Instead of the enriched monocyte fraction in the mentioned protocol, 5 × 10^6^ PBMCs/well were incubated on flat-bottom 6-well plates (Greiner) in RPMI medium for one hour at 37°C. Then, cells were washed with warm PBS to remove the non-adherent cells, enriching for adherent cells such as monocytes. Cells were incubated with 1, 5, or 10 μg/mL BCG (Danish strain, Statens Serum Institute), 2 μg/mL β-glucan, or RPMI control for 24 h, washed with PBS, and rested for five days in RPMI supplemented with 10% pooled human serum from healthy donors independent of the cell donors. After resting, cells were restimulated with 1 ng/mL *E.coli*-derived LPS for 24 h to determine cytokine production. For the telomerase activity assay or telomere length measurements, cells were gently scraped before any restimulation and processed further.

### Method details

#### PBMC stimulation

PBMCs from whole blood of study participants were isolated using Ficoll-Paque (GE Healthcare) density gradient separation and washed twice with phosphate-buffered saline (PBS). Cells were resuspended in Dutch-modified RPMI 1640 medium (Invitrogen), supplemented with 50 μg/mL gentamycin, 2 mM Glutamax (Gibco), and 1 mM pyruvate (Gibco). 5 × 10^5^ PBMCs/well were cultured in round-bottom 96-well plates (Greiner) and incubated for 24 h at 37°C with either RPMI control or 1 × 10^6^ CFU/mL heat-killed *S. aureus*. Supernatants were collected and stored at −20°C until analysis. IL-1β, IL-6, and TNF concentrations were measured using DuoSet ELISA kits (R&D Systems) according to the manufacturer’s protocol. Different time point samples belonging to a participant were measured on the same plate to minimize variation in the calculated cytokine fold changes.

#### Hormone measurements in plasma

Testosterone, androstenedione, dehydroepiandrosterone sulfate, and 17-hydroxyprogesterone levels in plasma samples from 300BCG participants before vaccination were measured by liquid chromatography with tandem mass spectrometry (LC-MS/MS) after protein precipitation and solid-phase extraction as described in detail previously.^58^

#### Telomere length measurements from whole blood

DNA from whole blood was isolated using QIAamp Blood kit (Qiagen), and concentrations were measured at 260 nm with a NanoDrop (Thermo Scientific) spectrophotometer. The average TL of the DNA samples was assessed using the Absolute Human Telomere Length Quantification qPCR Assay Kit (ScienCell) according to the manufacturer’s protocol. The kit includes a telomere-specific primer set (TEL) and a single-copy reference gene primer set (SCR) for data normalization. The reference genomic DNA sample provided with a known TL was used to calculate the average TL of the samples. The telomeric DNA was amplified and quantified by RT-qPCR (QuantStudio Real-Time PCR, Applied Biosystems). The average TL of the target sample per diploid cell was calculated as Reference sample TL x 2^-ΔΔCt^ where ΔΔCt = (Ct (TEL, target sample) - Ct (TEL, reference sample)) - (Ct (SCR, target sample) - Ct (SCR, reference sample)). TL per chromosome end was calculated by dividing the TL per diploid cell by 92.

#### Telomerase activity assay

Telomerase activity in equal numbers of control or BCG-trained cells was assessed using the Telomerase Activity Quantification qPCR Assay Kit (ScienCell) following the manufacturer’s protocol. Briefly, cells were lysed in the kit’s lysis buffer to release intact telomerase and incubated in the reaction buffer to allow the synthesis of new telomeric DNA. Subsequently, the telomeric DNA was amplified and quantified by RT-qPCR (QuantStudio Real-Time PCR, Applied Biosystems) using a telomere-specific primer set. Therefore, a lower Ct value for telomeric DNA amplification represents higher telomerase activity. Relative telomerase activity of one sample to another was calculated as 2^-ΔCt^ where ΔCt = Ct (target sample) – Ct (control sample).

#### RNA extraction and sequencing

Adherent PBMCs of 5 BCG-naïve healthy individuals were trained with 5 μg/mL BCG or RPMI control according to the *in vitro* trained immunity protocol mentioned above. At day 6, cells were harvested by scraping, centrifuged, and resuspended in 350 μL of Lysis Buffer RA1 from the NucleoSpin RNA kit (Macherey-Nagel) and saved at −80°C until RNA isolation. RNA was isolated using the NucleoSpin RNA kit following the manufacturer’s instructions.

Total RNA was used to prepare the RNA sequencing libraries using the KAPA RNA HyperPrep Kit with RiboErase (KAPA Biosystems). Oligo hybridization and rRNA depletion, rRNA depletion cleanup, DNase digestion, DNase digestion cleanup, and RNA elution were performed according to protocol. Fragmentation and priming were performed at 94°C for 6 min. First strand synthesis, second strand synthesis, and A-tailing were performed according to the protocol. For the adaptor ligation, a 1.5 μM stock was used (NextFlex DNA barcodes, Bio Scientific). The first and second post-ligation cleanup was performed according to protocol. A total of 11 PCR cycles were performed for library amplification. The library amplification cleanup was done using a 0.8x followed by a 1.0x bead-based cleanup. Library size was determined using the High Sensitivity DNA bioanalyzer kit, and the library concentration was measured using the dsDNA High Sensitivity Assay (Denovix). Paired-end sequencing of reads of 50 bp was generated using an Illumina NextSeq 500.

RNA sequencing data were processed using NEXTFLOW pipelines (version 21.04.3).^59^ Out of this, feature count tables were normalized and loaded to the Deseq2 tool (version 1.40.1) for differential expression analysis.^60^

### Quantification and statistical analysis

Data were assessed for normality using the Shapiro-Wilk test. For non-normal data, the Wilcoxon matched-pairs signed rank test was used for statistical comparison between two groups with paired samples such as different time points of the same participants. Mann-Whitney U test was used to compare two non-paired groups, males and females. For data following a normal distribution, paired t test was used to compare paired samples. Correlations were performed using Spearman’s rank correlation. *p* values below 0.05 were considered statistically significant. Statistical tests, significance degrees, sample sizes (n), and other relevant details are reported in the figure legends. Non-significant *p* values are reported in the figures. n represents the number of blood donors.

All analyses and plotting except for Figure 1 were performed on GraphPad Prism 8. For the RNAseq data (Figure 1), gene set enrichment analysis was performed using the gseGO function of the clusterProfiler package in R (4.1.3). PANTHER over-representation enrichment analysis was performed using the rba_panther_enrich function of the rbioapi package.^61^

### Additional resources

Details of the clinical trials referred to as the 300BCG study and the validation study can be accessed via the Dutch trial registry (https://onderzoekmetmensen.nl/) with reference numbers NL58553.091.16 and NL58219.091.16.

## Supplemental information

Document S1. Figures S1–S4

File S1. 300BCG telomere length

File S2. Validation study TL
