# Supplementary material for: Long-term effects of BCG vaccination on telomere length and telomerase activity
Source: iScience. 2025 Jul 18;28(8):113159. doi: 10.1016/j.isci.2025.113159 (PMC12341624; doi:10.1016/j.isci.2025.113159)
Supplement: Document S1. Figures S1–S4 [file mmc1.pdf]

iScience, Volume *28*

Supplemental information

Long-term effects of BCG vaccination on telomere length and telomerase activity

Ozlem Bulut, Valerie A.C.M. Koeken, Simone J.C.F.M. Moorlag, Charlotte J. de Bree, Vera P. Mourits, Gizem Kilic, Priya A. Debisarun, Marijke P.A. Baltissen, Joost H.A. Martens, Jorge Domínguez-Andrés, Leo A.B. Joosten, and Mihai G. Netea

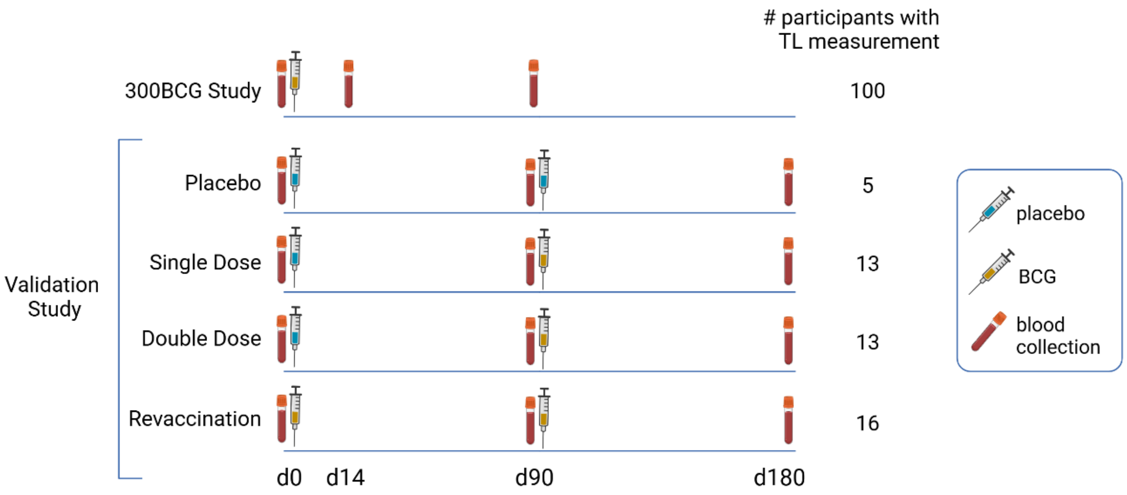

Figure S1. Vaccination and blood collection schedules of the studies.

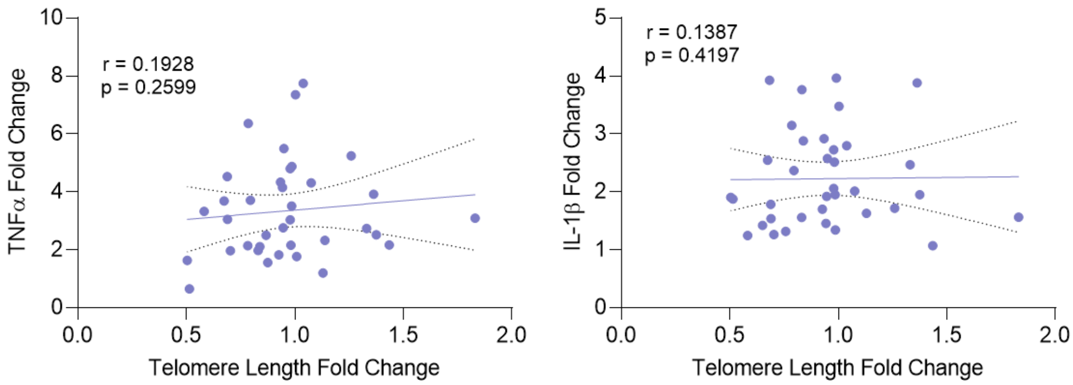

Figure S2. Correlations of improvement in TNF and IL1β responses with the telomere length change three months after BCG vaccination in trained immunity responders. Cytokines were measured upon 24 hours *ex-vivo* stimulation with *S. aureus*. Statistical analyses were performed using Spearman's rank correlation. r: Spearman's rank correlation coefficient.

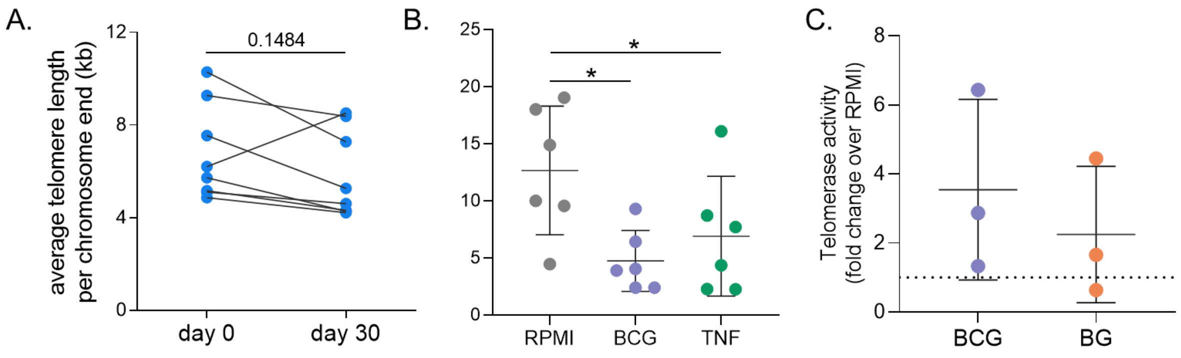

Figure S3. Telomere length and telomerase activity after training with non-BCG stimuli. A. Average telomere length per chromosome end in whole blood before and 30 days after MMR vaccination of healthy adult volunteers (n=8). B. Average telomere length at day 6 of *in vitro* training of healthy PBMCs with BCG or TNF (n=6). C. Telomerase activity relative to RPMI control at day 6 of *in vitro* training with BCG or β-glucan (BG) (n=3). The dashed line depicts the fold change of 1. Statistical analyses were performed using Wilcoxon matched-pairs signed rank test. * p≤0.05.

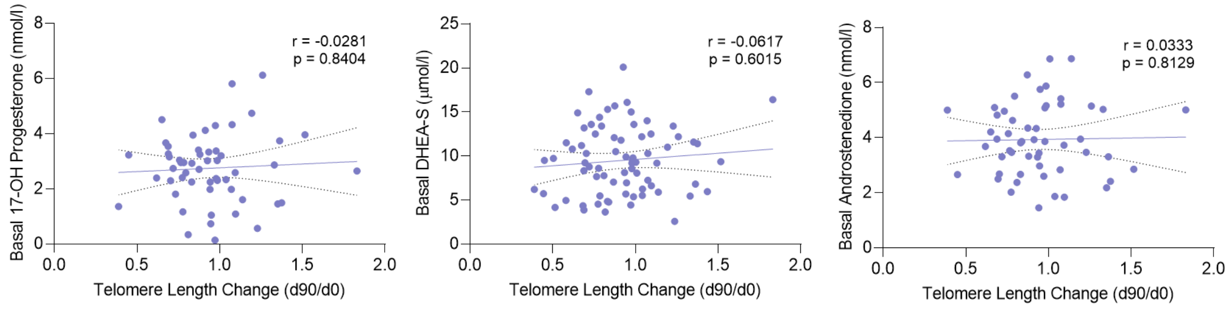

Figure S4. Correlations of basal hormone levels with the telomere length change three months after BCG vaccination. Statistical analyses were performed using Spearman's rank correlation. DHEA-S: dehydroepiandrosterone sulfate, r: Spearman's rank correlation coefficient.
